# Late onset postpartum psychoses

**DOI:** 10.1007/s00737-016-0680-y

**Published:** 2016-10-06

**Authors:** Ian Brockington

**Affiliations:** University of Birmingham, Edgbaston, Birmingham, B15 2TT UK

**Keywords:** Postpartum psychosis, 4–13 week onsets, Late postpartum onset, Weaning onset

## Abstract

It has been known since the eighteenth century that postpartum psychoses can begin several weeks after childbirth, not during the first fortnight. There are almost 400 non-organic episodes in the literature, starting more than 3 weeks after the birth; some of them are recurrent. The distinction of this disorder from early onset puerperal psychosis is supported by the distribution of onsets (which shows a steep fall after 14–15 days), survey data and the association with later pregnancies, not the first. Marcé believed that these late onsets were related to the resumption of menstruation. This is a hypothesis worth investigating.

## Introduction

Late onset postpartum psychosis was first described in the eighteenth century (Hoffmann 1721):

Four weeks after childbirth a 20-year old, rather melancholy and anxious by temperament, had a severe fright - she ‘saw’ the ghost of her long-dead mother. Three days later she became confused and started to rave. She was restless, talked day and night (mainly in rhyme), and ate and drank little, but had no fever. The infant was given to a wet-nurse, leaches were applied to her feet, and medicines given to bring on the menses; but her raving increased more and more, and she had to be restrained. It was 2 months before she calmed down. She immediately became pregnant again remained well.

Burns (1809) asserted that puerperal mania in some cases started several weeks after delivery. Marcé (1858) believed that it could start *either* immediately after the birth *or* several weeks later at the first menses; in separate enumerations, he gave ratios of 11/44 (1:4) and 12/60 (1:5) late/early postpartum psychoses; among his cases that meet my definition of psychosis, his figures were 10/29 (1:2.9). Fürstner (1875) supported this, stating that puerperal psychosis broke out *either* between days 10–12 *or* at 4–6 weeks, with a free period between, and two of his *hallucinatorische Irresein der Wöchnerinnen* had late onset. It is remarkable how little attention has been paid to this.

## Onsets of postpartum psychosis

Data on onset are often given in vague terms—“immediately after the birth”, “in a few days”, “in the second week”, *et cetera*. Focusing on those with more precise information, the day-to-day and week-to-week onset of non-organic episodes in the literature showed a sharp fall after 15 days, with few in the third week. This was confirmed in 155 from my series, which had better information (Brockington 2014). This suggested that the limit for early puerperal onset was not 6 weeks, or 4 weeks, or 3 weeks, but 15 days. Charted week-by-week Table 1 shows the onset of over 1400 episodes in the literature and 206 in my series, the totals are higher because cases with more approximate onset could be included.

**Table 1:** Week-by-week onset of non-organic postpartum psychoses

Week	Literature		My series			
			All cases		Bipolar/cycloids	
	Episodes	% fall	Episodes	% fall	Episodes	% fall
1	610		159		139	
2	405	34	59	63	45	68
3	92	77	11	81	6	87
4	106		6		4	
5	26		11		3	
6	73		10		4	
7	14		4		4	
8	82		5		1	

In both series, there is a steep fall from week 2 to week 3; if this rate of fall were continued, one would expect only 21 onsets in the literature, and only two onsets in my series, in week 4. In the literature, there are peaks in week 1, week 4, week 6, and week 8. In my series, which had relatively good data, there is a second mode at week 5–6, supporting Marcé’s idea. But this does not survive the study of bipolar/cycloid patients, which shows a long tail, but no second mode; it does, however, show the same sharp fall from week 2 to week 3, and, if continued, there should be only one case in the fourth week, and none thereafter.

Table 2 shows the month-by-month onset.

**Table 2:** Month-by-month onset

Month	Literature	My series
1	1213	235
2	195	30
3	80	8
4	61	4
5	34	3
6	35	2
7	19	3
8	19	2
9	19	2
10	13	1
11	6	nil

This shows relatively few episodes with onset more than 3 months after the birth. In my series, there were only 18 episodes, and only 3 bipolar/cycloids after 6 months and none after 8 months.

## 4–13 week onsets

In the literature, 447 patients had onset in this time frame. There were only 48 organic psychoses (11 %), much fewer than after abortion, during pregnancy or in the early puerperium (all over 30 %). Almost all were infective delirium. The remaining 399 mothers had non-organic episodes, including 326 single and 73 recurrent cases (409 episodes). The rate/trimester (531/trimester) is one tenth that of early postpartum onset (5629/trimester), but much higher than those starting during pregnancy (146/trimester) or after abortion (108/trimester). The ratio of 4–13 week onsets to early postpartum onset is 1 to 3. In my series, there were only 41 episodes with 4–13 week onsets, and of these only 19 were bipolar/cycloid; the ratio of 4–13 week to early postpartum onset was 1 to 7, lower than in the literature.

### Parity

Only 109 (32 %) were *primiparae*. Restricting the analysis to bipolar/cycloid patients in my series the mean parity was 2.19, with 38 % *primiparae*. This compares with a mean of 1.58, with 58 % *primiparae* in early onset postpartum episodes. There is confirmation of this higher parity in a Danish survey of 750,000 women: in *primiparae*, the highest relative risk of psychiatric disorder (not just psychoses) occurred 10–19 days postpartum (RR 8.7), but, when the first psychiatric episode followed the second birth, it was 60–89 days postpartum (RR 2.7) (Munk-Olsen et al. 2014).

### Recurrent cases

There are eight cases in the literature, and four in my series, with two 4–13 week onsets and no other reproductive episodes. One mother had three episodes, but only a vague description—“She became insane some weeks later, each time with various delusions” (Gilmore 1892). In addition to multiple episodes limited to this time frame, one mother had seven postpartum episodes, including three late onsets—at 4 weeks, 2 months and 3 months, with the rest of unknown onset (Ideler 1851). This Danish patient (Holm 1874) had three early and three 4–13 week postpartum onsets:

A labourer’s wife, aged 19, gave birth to her second child in 1834; on day 3 she became manic, and remained ill for 7 months. In 1837 she gave birth for the 3rd time, and breast-fed for 2 months, at which point she became manic for 11 months. In 1839 she gave birth for the 4th time and breast-fed for 1 month, when she became manic for an entire year. In 1841 she had her 5th child, whom she breast-fed for 2½ months; when she weaned the child she became manic for 13 months. In 1844, after her 6th delivery, she failed to lactate, and immediately became manic, lasting 8 months. In 1847 she gave birth to her 7th child, who died on the 8th day; she broke out into mania, less severe but chronic with remissions. In 34 years’ observation, she also had four episodes unrelated to childbearing.

The six postpartum episodes, all within the first trimester, cannot have been sporadic because she had only four unrelated episodes in 104 non-reproductive trimesters (*p* = 0.0001); but the postpartum episodes started in two separate time frames.

There are many recurrent cases with a concurrence of 4–13 week onsets and other onsets—31 in the literature and 27 in my series (combined total 58); 31 of these were associated with early postpartum onset, 17 with prepartum onset, 15 with onset after 3 months and 5 with post-abortion onset.

## Late postpartum onset

Onsets more than 3 months after childbirth are much less common—226 published cases, spread over 9 months not 10 weeks, a rate/trimester one third that of 4–13 week onsets. Eleven were organic psychoses, 157 single non-organic episodes, and 58 recurrent cases.

### Recurrent cases

Seven mothers had two or more episodes with onset later than 3 months, with a total of 24 episodes (Hurt 1911; Masieri 1925; Mitkus 1927; Rabinowitsch 1928; Ménaché 1929; Fumarola 1935; Blinov et al. 1936). There are three interesting cases. One mother (Ménaché 1929) had four episodes of acute mania, with excitement, logorrhoea, clang associations and eroticism, with admission to hospital 2, 5, 6, and 6 months after her four births; no other episodes were recorded in 8 years (*p* = 0.03). Two mothers had a rather similar brief recurrent hallucinatory psychosis (Hurt 1911; Mitkus 1927):

A French woman gave birth to six children. Five months after her 3rd birth she ‘heard’ people walking about in her bedroom - she opened the window and called for a ladder to make her escape. Admitted to hospital, she was in a state of terror with severe hallucinations, including left-sided hallucinations of hissing, barking, mewing and ticking clocks. She soon recovered and was discharged after 18 days. She gave birth again and, after 13 months lactation, developed a similar episode with terrifying auditory, visual, gustatory, olfactory and somatic hallucinations. Most of the time she was mute and stuporose, but she suddenly emerged to fight off a wild boar, and a man who was trying to shove her into a stove. She rapidly recovered and was discharged after 28 days. Three months after the 6th child was born, she had a similar episode that lasted 15 days, and, 1 year later, long after weaning, she had the fourth episode that lasted 3 weeks, followed, 7 months later by a 5th episode with mutism and visual hallucinations of intruders and flames, for 1 day only.

A gentle, hard-working, and exemplary Polish housewife had her first mental illness after 4–5 months of lactation. This took the form of confusion with disorientation, stupor and later excitement. She saw the judgment of God, the Virgin Mary and dead people everywhere - everyone had died, including her brothers, one of whom was buried as a woman wearing stockings, covered with a towel; she heard their voices. She gave birth 11 times, and had similar episodes (with similar content) in the lactation phase after the 6th, 9th and 11th births, always with complete recovery.

With the other five recurrent cases, with 2-5 episodes each, these support the action of some unknown but specific late postpartum factor.

There were 45 cases in the literature and 10 in my series with complex associations (a combined total of 55 cases); 24 were associated with early postpartum onset, 15 with 4–13 week onsets, 15 with prepartum onsets and only one with post-abortion onset.

## Weaning onset

This is a special instance of late postpartum onset, and includes some cases starting more than 12 months after childbirth (Marcé 1856, 1858; Cortyl 1877; Martin 1880). Excluding an eighteenth century case (Williardts 1770), in which ‘manic fury’ began 8 weeks after the birth when ‘the breasts were hanging and empty’, the first (a recurrent) case was reported by Esquirol (Esquirol 1819):

A 30-year old was the mother of three infants. Two days after ‘incautiously’ weaning her 4th child, she suffered *délire général* with religious ideas, and recovered after 4 months. At 39 she gave birth to her 5th child: seven months later, the day after weaning, she developed a rash and *délire* and imaginary fears; she spent 20 months in the Salpêtrière in a state of hopeless melancholy with religious terrors.

Excluding cases with a birth interval less than 3 months (when other puerperal factors could compete), the literature consists of 32 cases—18 single and 14 recurrent, including 4 with two or more weaning episodes. In most, the interval between weaning and onset was not stated, or only in vague terms (‘at the time of weaning’, ‘shortly after weaning’), and often there was little information: for example, there was this tantalizingly brief description of a mother with multiple episodes (Loiseau 1856):

A woman always became mad in the third month of lactation. She had seven or eight children. With each new birth she insisted on breast-feeding her child, and every time, in the third month, her milk was suppressed and mania broke out.

Marcé had eight patients with weaning onset, six of whom had suckled their infants for a long time (10–21 months). This is one of his cases:

A 26-year old, working as a domestic at the Salpêtrière, breast-fed her 2nd child for 14 months. After an argument with her boss she abruptly weaned the child, and, 3 days later, became overactive, sleepless and incoherent. Admitted to the same hospital, she was excited and overtalkative. Three days later she menstruated for the 1st time since the birth. She recovered and was discharged well after only 8 days.

This mother may have had three episodes (Révolat 1847):

A 30-year old, while breast-feeding her 3rd child, had a scare that one of her children had been run over by a carriage; although this was not true, she immediately developed a *folie*, which lasted 2 years. The author did not know whether suppression of her milk came before, after or simultaneously with this event. Five years later, after breast-feeding her next child for a year, she weaned it abruptly and immediately became manic for 4 months. The same happened with the next pregnancy – the psychosis started at the moment of weaning, and lasted 18 months. In the course of 8–9 years she had one other episode unrelated to childbearing.

Most of the examples were published in the nineteenth century, but this case was recently reported from New Zealand (Joyce et al. 1981):

A 31-year old, whose father probably committed suicide, breast-fed her 1st child for 12 months; her milk had been drying up but the child appeared to initiate the weaning. Immediately afterwards, she developed a manic episode that lasted 6 weeks and was followed by depression lasting 4–5 months. Two years later, within a week of weaning her 2nd child, aged 6 months, she developed another psychosis, with perplexity, insomnia, restlessness, over-activity, over-talkativeness, pressure of speech and flight of ideas. She recovered within 3 weeks.

In my series, I had no convincing case of weaning onset. It seems possible that, in Victorian times, mothers, with less access to safe supplementary feeding, breast-fed for longer. But in third-world countries, breast-feeding rates are still high, and, if this is the explanation, one would expect to see weaning onsets.

## Discussion

There are several reservations about these data:

- They are all based on records of episode onset, which is difficult to determine precisely
- Delayed hospitalization, rather than delayed onset, is possible. The fact that there are proportionately more cases in the literature than in my series (and there is No impediment to the admission of late onset cases to British mother-and-baby units) is in line with this explanation.
- Onset in the second month could be due to the development of depressive psychoses later than acute mania; but although depressive psychoses were more frequent in the 4–13 week onset group, they were still a small minority; so most mothers who become ill in this time frame are not suffering from depression, triggered by the birth but slow to develop or reach psychiatric care.
- It is possible that late onset does not reflect the activity of a late trigger, but just the length of the pathogenetic process and late appearance of symptoms, that is, a long tail in the skewed distribution of onsets. But the shape of the distribution in Table 1, which is bimodal in my series, and shows a sharp fall after the second week in all three groups, is against this explanation. It seems unlikely that a trigger that has maximum effect in the first 15 days can incubate a psychosis that erupts a month later.

Marcé’s intuition linked these late onsets to the resumption of menstruation. He wrote (Marcé 1862),

The first postpartum menses exercises, on the development of puerperal insanity, an influence that Baillarger was the first to notice, and which my observations confirm *beyond doubt* [my italics]: of 44 mothers who developed puerperal psychosis, and who did not lactate, 11 became ill in the 6th week, precisely at the return of the menses. Sometimes the psychosis preceded the menses by 5–6 days, but it usually began at the onset of bleeding or during menstrual flow. I have also seen it break out when the menses were expected, but failed to appear. Mothers, who breast-feed for some months, become ill after weaning, very often at the moment the menses reappear after a long interval.

If Marcé’s ideas are correct, these late onset cases should not be termed ‘puerperal psychoses’—they are menstrual psychoses. Although Marcé made these assertions over 150 years ago, they have never been taken up, and never investigated. Of course, they should be doubted, but not ignored.

This explanation extends to onsets later than 3 months in lactating women; indeed, his weaning cases suggest it could apply after the first postpartum year. We now know that, in mothers who do not breast-feed, ovulation does not occur until after about 5 weeks (Perez et al. 1972), and menstrual bleeding (ovulatory or non-ovulatory) about 2 weeks after that. In lactating mothers, menstruation returns before weaning, especially in those who supplement breast-feeding. A review concluded that amenorrhoea persisted for about 9 months (Chao 1987).

Until recently, his menstrual hypothesis was the only one on the table. But it has now been suggested that auto-immunity could have a role (Bergink 2013). This is based on a single case of early onset puerperal psychosis, with the development of auto-immune thyroiditis 3 months later; this thyroid disorder, affecting about 5% of women, was discovered in 1956 (since when about 1000 detailed cases of postpartum psychosis end sentence at have been published), which affects about 5 % of women. The hypothesis is supported by:

- The general proposition that, in pregnancy, antibody titres decrease to protect the foetus, and rebound in the puerperium, with a flaring up of pre-existing auto-immune disorders.
- Evidence that bipolar disorder is associated with immune/inflammatory dysfunction, including auto-immune thyroiditis (Kupka et al. 2002); but a Danish population study involving nearly 1000 cases of bipolar disorder and over 3.5 million births found only one association – pernicious anaemia (Eaton et al. 2010).
- Bergink and her colleagues in Rotterdam found that 6/31 mothers with puerperal psychosis had evidence of auto-immune thyroid disease at 4 weeks, and nine at 9 months postpartum, both being more frequent than 117 controls (*p* = 0.02). This finding has not yet been replicated by another group.

It is not plausible that a disorder, with no prior history, triggered during parturition, could be an auto-immune ‘flare up’, but this could apply to late onset postpartum psychoses. There are relevant two cases in the literature (Bokhari et al. 1998; Stowell and Barnhill 2005). This is Bokhari’s:

Eleven weeks after her 3rd child was born, a 29-year old developed insomnia, weight loss and fatigue intolerance. She appeared confused and was disorientated in time and place. She heard Jesus talking to her, and also had visual hallucinations. She believed she was pregnant with the Christ child, and would be killed by hospital staff. She had thyrotoxicosis, associated with thyroiditis. Her psychiatric symptoms improved concurrently with its treatment.

Her psychosis, with organic features, responded to thyroid treatment; it could be caused by thyrotoxicosis, not auto-immunity, since at least ten thyrotoxic psychoses have been published (Brockington 2016).

Although there are reservations, It seems best provisionally to accept Marcé’s proposal that late onset episodes are a distinct phenomenon, and should not be included in research on ‘puerperal psychosis’. Once they have been separately diagnosed, there is an opportunity to research the role of breast-feeding, auto-immunity and the menstrual process.
